# Abstract of the Proceedings of the Buffalo City Medical Association at Its Regular Meeting, Held Tuesday, Ang. 1st, at the Office of Dr. Hamilton

**Published:** 1854-09

**Authors:** Sanford B. Hunt


ART. IV.—*Abstract of the Proceedings of the Buffalo City Medical Association at its Regular Meeting, held Tuesday, Aug.* 1*st, at the office of Dr. Hamilton.*

The President, Dr. Newman, in the chair.

*Present*—Drs. Eastman, Gould, Wilcox, Congar, Nelson, Rochester, Jeyte, Gay, Hamilton, Hunt, Strong, and Wyckoff.

Minutes were read and approved.

Dr. Hunt gave a sketch of the hygrometric conditions of the month of July, for the result of which reference is made to the Buffalo Medical Journal for September.

Dr. Hamilton remarked, that though we have no definite knowledge of the ultimate causes of cholera, we possess as accurate knowledge of its exciting and immediate causes, as of any other disease. The peculiar haunts of the disease, its constant association with miasmata of some sort, are evidences of this. He had no doubt that tellurial influences were the true exciting cause, and that the humidity accompanying a high dew-point aided, at once, their development, their solution and dispersion in the atmosphere, and their absorption into the system. The occurrence of cholera in winter in Russia is no contradiction of this. The houses of the Russians are heated by stoves, and so constructed as to avoid ventilation, so that the air within them is extremely hot and moist, and they live very much within doors.

Dr. Congar mentioned that cholera sometimes occurs in ships at sea during calms.

Dr. Nelson said that inattention to dryness between decks is the principal cause of disease on ship-board. Vessels leave port in a very filthy condition, incident to getting emigrants or cargo on board. The between-decks are then usually cleaned by the free use of water, and "damp swabbing," making them very moist. If typhus fever, or other disease, breaks out, lime is scattered about deck to absorb the moisture.

Dr. Hamilton—There was no cholera at Suspension Bridge in '49 and '51. At those times no excavations were being made, and though many emigrants passed through with cholera among them, and died along the route, no cholera occurred among residents. During the past winter and spring very extensive excavations have been made, and standing pools of water formed. Such a pool existed behind the Bellevue House, in which five deaths occurred. No cases originated except in the immediate neighborhood of this excavation.

Dr. Congar—Heat alone, without humidity, seems to act as a cholera cause at the South.

Dr. Hunt—During the prevalence of the yellow fever in New Orleans last year, the dew-point was very high; there were daily showers followed by hot sun. In a zone reaching from the Mississippi to the Atlantic, in the latitude of Tennessee, there was a period of heat with low humidity, the heat reaching 100°, which was higher than anywhere on the gulf. No sickness accompanied it. In Central America the climate is both humid and sickly.

Dr. Newman—There are 25 houses on Mortimer street in this city, in 17 of which there were 50 cases of cholera, of which 20 died. The inhabitants attributed their sickness to a well which supplied them water. This well is bored 45 feet in the rock, and tubed with copper. The pump had been out of order, and the water was not used for some days. When it was in use again the people who used it became sick, as they thought, from the water. Cholera occurred however in two or three houses where this water had not been used. A sewer in Genesee steet is laid in the rock so that its contents must communicate with the wells. In Mortimer and Cherry streets, where cholera has most prevailed, there is no proper sewerage. Some of the inhabitants bore through the rock till they reach a seam, and then connect their drains with this boring. Such sewerage must poison the water of the wells.

Dr. Nelson—No city (Niagara) water is supplied to those streets where cholera has prevailed. On Monroe street the wells are sulphurous. The people have diarrhœa from their use. Is the water on Mortimer street sulphurous?

Dr. Rochester—Cholera has never prevailed at the sulphur springs, such as Avon, Clifton, or Sharon. At a house on Michigan street, where sulphur water was used, an old lady had cholera. Her nurse was next taken, then her son, and finally her husband. Dr. R. finally found that a neighboring privy had communicated its smell to the cistern of the family, and that a cess-pool close by had also the peculiar smell of the privy, and that the house was frequently impregnated with it.

Dr. Wilcox inquired whether any member had witnessed cases of cholera without discharges from the bowels. If such cases occurred, it was likely to destroy the confidence of community in the easy management of cholera in its early stages.

Dr. Rochester mentioned such a case as within his observation while residing in New York. Such an one also occurred in Dr. Flint's practice in this city in 1849.

Dr. Congar had recently lost a relative in Chicago from cholera without any discharges.

Dr. Hunt had seen some cases at Suspension Bridge in which there was no premonitory diarrhœa. In many cases the amount of discharges was not very great. In a case at Suspension Bridge, he supposed the man dying. From a suggestion received some time since from Dr. Rochester he decided to apply the wet sheet. Very much to his surprise the man recovered. Subsequent cases, perhaps a dozen in number, did not confirm the promise of the first case. He could trace no direct curative influence from its application; but, in all cases it relieved the cramps, and removed the blueness of the skin. If the patients were as well "packed" as they should be, it promoted the warmth of the surface. It was very grateful to the patient.

Dr. Rochester made no claim to originality, in this suggestion, but he thought the application might be serviceable, because the skin might thus absorb water, and restore the fluidity of the blood, and the secretions of the kidneys. He would combine with this treatment large doses of Spirits Etheris Nitrosus.

Dr. Congar inquired of the results of the use of calomel. In a former epidemic he had given large and repeated doses. He had had reason to regret this course, and thought that as much harm as benefit resulted from such heroic treatment. He would now confine his treatment to small doses of calomel, with a view to correcting the secretions; disregarding the alleged sedative effect of the large doses.

Dr. Nelson related a case in which diarrhœa had been very persistent under opiates, which yielded readily to this mixture:

℞ Tinct. Capsicum, ℨ ij.

Sulph. Acid Dilut., ℨ ij.

Camphor Water, ℥ ij. M.

Give a tea-spoonful for a dose, every 15 minutes, with 4 or 5 drops of laudanum.

Dr. Gay related a case in which small doses of calomel and morphine were persistently rejected. He then gave a thirty gr. dose of calomel, with an equal quantity of chloride of sodium; which was retained, and the discharges controlled at once. In reply to an inquiry, he said he had frequently given calomel in this combination, without discovering any chemical reaction.

Dr. Wilcox was not in the habit of giving large doses of calomel. He sometimes gave 10 grs. but usually only small doses in combination with full doses of morphine, repeated as necessary. The greatest error in the management of cholera, as he thought, was in the constant perturbation of the patient by frictions. He regarded rest as the leading indication. All the adjuvant treatment, such as mustard and hot applications, should be applied so as to avoid fatigue. He found the mustard over the stomach the most efficient check upon the vomiting.

Dr. Rochester—Sometimes gives the Acetas Plumbi with morphine. As a diffusible stimulant he prefers Carb. Ammon, and Sulph. Ether, to brandy.

Dr. Newman found that gin was much better retained than brandy, and seemed a kinder stimulus. The Sisters of Charity at the Hospital had great faith in blistering. They had lost no one upon whom they had succeeded in getting a good blister. Probably the blister afforded an index to the vitality of the skin, and in cases with a decidedly fatal tendency it would not draw.

Dr. Hamilton read a paper giving an account of the appearances of some cases of Varicella he had seen at Hornellsville; about the diagnosis of which there had been much dispute. It was agreed that they could not have been Variola.

Society adjourned to meet at Dr. Hunt's office.

SANFORD B. HUNT, Secretary.

